# Content and face validity of Workplace COVID-19 Knowledge & Stigma Scale (WoCKSS)

**DOI:** 10.1186/s12889-023-17614-3

**Published:** 2024-03-21

**Authors:** Izyan Hazwani Baharuddin, Nurhuda Ismail, Nyi Nyi Naing, Khalid Ibrahim, Siti Munira Yasin, Megan S. Patterson

**Affiliations:** 1 Department of Public Health Medicine, Faculty of Medicine, Universiti Teknologi MARA Selangor Campus, Jalan Hospital, 47000 Sungai Buloh, Selangor Malaysia; 2 Faculty of Dentistry, Universiti Teknologi MARA Selangor Campus, Jalan Hospital, 47000 Sungai Buloh, Selangor Malaysia; 3 Faculty of Medicine, Medical Campus, Universiti Sultan Zainal Abidin, Jalan Sultan Mahmud, 20400 Kuala Terengganu, Terengganu Malaysia; 4 Department of Health Behavior, 1226 Texas A&M University, College Station, , Texas, 77843 USA

**Keywords:** COVID-19, Workplace, Knowledge, Stigma, Manufacturing industry, Content validity, Face validity, WoCKSS

## Abstract

**Background:**

The COVID-19 pandemic has led to fear, rumours, and stigma, particularly against those infected with the virus. In Malaysia, the manufacturing industry is particularly vulnerable to COVID-19 clusters, making it critical to assess stigma attitudes among workers. To address this issue, The Workplace COVID-19 Knowledge & Stigma Scale (WoCKSS) was developed specifically for use in the manufacturing industry which served as the sample population for testing this scale. It was developed in the Malay language to ensure alignment with the local context. This study examines the content and face validity of WoCKSS, which can help assess the level of knowledge and stigma associated with COVID-19 among workers.

**Methods:**

The WoCKSS was developed with 20 and 31 items for knowledge and stigma domains, respectively, based on an extensive review of COVID-19 literature. Content validation was conducted by four experts using a content validation form to assess the relevancy of each item to the intended construct. Content Validity Index (CVI) was calculated to measure the agreement between the experts on the relevance of each item to the intended construct. Face validation was then conducted by randomly selecting 10 respondents from the manufacturing industry, who rated the clarity and comprehension of each item using a face validation form. The Item Face Validity Index (I-FVI) was calculated to determine the clarity and comprehension of each question, and only items with an I-FVI ≥ 0.83 were retained.

**Results:**

The WoCKSS achieved excellent content validity in both knowledge and stigma domains. Only 19 items from the knowledge domain and 24 items from the stigma domain were retained after CVI analysis. All retained items received a CVI score of 1.00, indicating perfect agreement among the experts. FVI analysis resulted in 17 items for the knowledge domain and 22 items for the stigma domain. The knowledge domain achieved a high level of agreement among respondents, with a mean I-FVI of 0.91 and a S-FVI/UA of 0.89. The stigma domain also showed high agreement, with a mean I-FVI of 0.99 and a S-FVI/UA of 0.86.

**Conclusion:**

In conclusion, the WoCKSS demonstrated high content and face validity. However, further testing on a larger sample size is required to establish its construct validity and reliability.

## Introduction

The COVID-19 pandemic has brought unprecedented global challenges, reshaping societies, economies, and health systems worldwide [1, 2]. The virus, known for its ease of transmission through respiratory droplets, has presented a spectrum of symptoms ranging from mild to severe, encompassing fever, sore throat, fatigue, cough, and dyspnoea [3–5].

During the COVID-19 crisis, Malaysia implemented a Movement Control Order (MCO) to curb the spread of the virus, resulting in the closure of numerous sectors [6]. This measure significantly impacted the Malaysian economy, causing substantial production disruptions due to forced business closures and workers' inability to commute to work [7]. The COVID-19 lockdown also markedly altered consumption patterns in Malaysia, with panic among consumers and firms distorting usual consumption patterns and creating market anomalies [7]. While serving as a cornerstone of the nation's economy, the manufacturing sector persevered in its operations throughout the pandemic, navigating global supply chain disruptions and occasionally facing closures when clusters emerged [8]. However, due to their persistent presence in the workplace, employees in the manufacturing sector are at a higher risk of contracting and spreading the virus [9, 10]. Operations within this sector inherently involve close proximity and prolonged contact among workers on production lines and in crowded factory settings, further heightening the risk of COVID-19 transmission in the workplace [11]. This is substantiated by data indicating that in Malaysia, 91% of emerged COVID-19 clusters occurred within workplaces. Among these clusters, factories accounted for a substantial 80%, with an additional 11% originating from construction sites [12].

The pandemic has led to stigmatization, where individuals faced discrimination, social isolation, and psychological distress, further pandemic’s intensifying the impact on mental health [13, 14]. Those infected with the virus have been labelled as "intentional murderers" [15] and "super spreaders" [16]. Consequently, this perception has translated into stigmatization and discrimination against manufacturing employees, with a particular impact felt on the factory floors [17]. This discrimination has been reported in Kashmir, where the COVID-19-affected workers are not only discriminated against in the place of work but also in the community and at the place where the factory is situated [18].

Stigmatization within the manufacturing sector can have profound effects. These effects can extend to their personal lives and interactions outside of work. Employees encounter discrimination stemming from stigma, leading to impediments to basic needs, insult, blame, defamation, and job loss in various countries [19]. The impact of stigma could be significant, as it could discourage people from adhering to public health measures, seeking medical care, or disclosing their infection status [10]. Therefore, addressing COVID-19 stigma has become increasingly important during this post-pandemic era,

Therefore, understanding the level of knowledge and the degree of stigma associated with COVID-19 in the workplace is necessary for implementing effective intervention programs that can minimize the spread of the virus.

Several instruments have been developed to assess COVID-19 stigma, each tailored to specific contexts and target populations. For instance, the COVID-19 Public Stigma Scale (COVID-PSS) was developed in Thailand, specifically designed for use among the general public [20]. This 10-item tool underwent rigorous testing to establish its validity and reliability, including exploratory factor analysis (EFA), confirmatory factor analysis (CFA), and Cronbach's alpha analysis [18]. Similarly, the COVID-19 Stigma Scale was developed and validated among Egyptian physicians using the English language. This 16-item scale also underwent thorough psychometric testing and was found to be valid and reliable based on EFA, CFA, and Cronbach's alpha analysis [21].

However, it becomes evident that existing scales, tailored for the general public and medical professionals, may not effectively address the specific needs of Malaysian manufacturing sector, especially since the previous tools was not validated in Malay language. Recognizing this gap, the Workplace COVID-19 Knowledge & Stigma Scale (WoCKSS) was developed as a specific tool tailored for assessing COVID-19-related knowledge and stigma within the Malaysian manufacturing sector. Unlike other scales, which are designed for general populations or healthcare professionals, WoCKSS is uniquely positioned to address the specific challenges and dynamics within the workplace. It is designed in the Malay language, the national language of Malaysia, ensuring that it resonates effectively with the target population of manufacturing employees and filling a critical gap in the literature. While other scales serve more general purposes, WoCKSS hones in on the unique issues faced by manufacturing workers in Malaysia, offering a reliable instrument to measure knowledge and stigma related to COVID-19 within this context.

Therefore, the aim of this manuscript is to develop the Workplace COVID-19 Knowledge & Stigma Scale (WoCKSS), a tool for assessing knowledge and stigma related to COVID-19 in a workplace setting, and to evaluate its content and face validity.

## Methods

### Scale development

The workplace COVID-19 Knowledge and Stigma Scale (WoCKSS) was developed in two (2) domains: knowledge, and stigma; with 20 and 31 items, respectively. The items were developed in the Malay language after conducting a qualitative review of the literature on COVID-19 knowledge and stigma. The literature search included sources from Scopus, Web of Science (WOS), and Google Scholar, aimed at exploring relevant literature. The search terms included "COVID-19", “knowledge”, "stigma", "questionnaire", "development", and "validation". While no specific inclusion and exclusion criteria were applied, the intent was to explore the availability of questionnaires related to COVID-19 knowledge and stigma in the workplace, with a particular focus on the manufacturing industry. It's important to note that the review focused on gaining a general overview of the subject matter rather than conducting a systematic, comprehensive review with specific search criteria. The findings from this exploratory search played a pivotal role in shaping the development of WoCKSS, contributing valuable insights that led to a nuanced understanding of the constructs relevant to the manufacturing workforce.

The stigma domain was especially developed based on three constructs, namely stereotype, prejudice, and fear following the COVID-PSS [20].

### Content validation

To ensure the content validity of the workplace COVID-19 knowledge and stigma scale, a thorough content validation process was conducted. The selection of experts for this process was carried out with careful consideration. A panel of four experts was carefully selected based on their diverse qualifications and expertise in relevant domains. The panel comprised experts with backgrounds in public health, including epidemiology and biostatistics, and family and community health. Additionally, a microbiologist and a primary care medicine expert enriched the multidisciplinary composition of the panel. The expert in epidemiology and biostatistics brought a wealth of knowledge in quantitative research methods and statistical analysis, ensuring methodological rigor in the scale development process. Another public health expert with a focus on family and community health contributed valuable insights into the socio-ecological aspects relevant to workplace settings, enhancing the scale's applicability to diverse community contexts. The microbiologist provided specialized knowledge in microbiology, offering critical perspectives on infectious diseases and microbiological considerations. This expertise enriched the content validation process, emphasizing the biological aspects of COVID-19 within the workplace context. The primary care medicine expert, bringing a crucial clinical perspective to the panel, ensured a holistic approach to the validation process, considering the practical implications of COVID-19 knowledge and stigma within primary care settings. While their direct experience with COVID-19-related scales may be limited, their diverse and comprehensive qualifications, coupled with their close involvement with COVID-19 within their respective fields of expertise and their experience in developing and validating scales across various health-related domains, add substantial credibility to the validation process.

Prior conducting the content validation, a content validation form was prepared, which consisted of a list of items and a rating scale. The form was designed to assess the relevancy of each item in the scale to the intended construct, i.e., workplace COVID-19 knowledge and stigma. The rating scale ranged from 1 to 4, with 1 being not relevant and 4 being highly relevant. The experts were informed about the purpose of the scale and the content validation process. They were asked to review the domain and items and provide comments on their relevance, clarity, simplicity, and ambiguity. Each expert provided a score on each item based on their relevancy to the intended construct.

The Content Validity Index (CVI) was then calculated for each item. The CVI is a widely used method to assess the content validity of a scale. It is calculated by dividing the number of experts who rated an item as 3 or 4 by the total number of experts. For Universal Agreement (UA), a score of '1' is assigned to the item that achieved 100% agreement among experts, while '0' is assigned to items that did not achieve 100% expert agreement. The mean I-CVI was also calculated by taking the sum of the Item Content Validity Index (I-CVI) scores for all items and dividing by the total number of items. The I-CVI is the proportion of experts who rated an item as relevant.

The Scale-level Content Validity Index/Unweighted Average (S-CVI/UA) and Scale-level Content Validity Index/Average (S-CVI/AVE) were also calculated for both domains. The S-CVI/UA represents the proportion of items that achieved a CVI of 1.0, while the S-CVI/AVE represents the average CVI across all items. The S-CVI/UA was calculated by dividing the number of items rated as "relevant" by all experts by the total number of items. The S-CVI/AVE was calculated by summing all the I-CVIs of the items that were rated as "relevant" and dividing by the total number of items. These calculations were performed to ensure that the scale had adequate content validity. Items with a CVI < 1 were considered problematic and were discarded [22–24].

### Face validation

To further ensure the validity of the workplace COVID-19 knowledge and stigma scale, a face validation process was conducted. The purpose of this process was to assess the clarity and comprehension of the items and ensure that the respondents interpreted the items in the same way as the scale developers intended [25]. Prior to conducting the face validation, a face validation form was prepared containing only the items retained after content validation. The form was designed for the respondents to rate the clarity and comprehension of each question on a 4-point Likert scale, ranging from 1 (not clear/comprehensible) to 4 (very clear/comprehensible).

A multistage random sampling technique was used to select 1 company, a paper manufacturer, from the manufacturing industry. Due to logistical constraints, this choice was made to ensure a feasible and effective face validation process. 10 respondents from the selected company were randomly invited to participate in the study. The face validation process was conducted by distributing the face validation form to the selected respondents through their management office. The respondents were asked to read each question and rate its clarity and comprehension based on their interpretation. They were also asked to provide comments on any unclear or ambiguous items.

After the face validation process was completed, the items were reviewed for clarity and comprehension. The proportion of respondents who rated each question as clear and comprehensible (scores of 3 or 4) were calculated to determine the Item Face Validity Index (I-FVI) for each question. The I-FVI was calculated by dividing the number of respondents who rated the question as clear and comprehensible by the total number of respondents. The Mean I-FVI was then calculated by taking the average of the I-FVI values for all items [26].

The Scale Face Validity Index (S-FVI/UA) was calculated by dividing the number of items that achieved an I-FVI value of 3 or 4 by the total number of items. The S-FVI/UA provides an overall measure of the scale's face validity. Additionally, the S-FVI/AVE was calculated by taking the average of the I-FVI values for all items. This provides a more conservative estimate of the scale's face validity since it takes into account the possibility of chance agreement among respondents. Finally, the Proportion Relevance was calculated by adding the I-FVI values for each question and dividing the sum by the total possible I-FVI values. This provides an indication of the proportion of items in the scale that are relevant to the construct being measured. The FVI value ranged from 0 to 1, with a score of 1 indicating perfect clarity and comprehensibility. Items with an FVI < 0.83 were discarded, as they were not considered clear and comprehensible [26, 27].

### Data analysis

Demographic characteristics were analyzed descriptively using IBM SPSS Statistics version 29. Calculations for content validation and face validation were executed in Microsoft Excel.

For content validation, calculations were performed for Item Content Validity Index (I-CVI), Universal Agreement (UA), Mean I-CVI, Scale-level Content Validity Index/Unweighted Average (S-CVI/UA), and Scale-level Content Validity Index/Average (S-CVI/AVE).

For face validation, Item Face Validity Index (I-FVI) was determined, and Universal Agreement (UA) was applied to calculate Scale-level Face Validity Index/Unweighted Average (S-FVI/UA) and Scale-level Face Validity Index/Average (S-FVI/AVE).

The decisions concerning item retention were primarily guided by the quantitative scores obtained from these analyses, ensuring a rigorous and standardized evaluation of each item's quality.

## Results

### Content validation

The results for content validity of the WoCKSS for the manufacturing industry are presented in Tables 1 and 2 for knowledge domain and stigma domain, respectively. The experts rated each item on a four-point scale, ranging from 1 (not relevant) to 4 (highly relevant). The number of experts who rated each item as a 3 or 4, which indicates relevance, is reported in the "Number in agreement" column. From an initial set of 20 items in the knowledge domain and 31 items in the stigma domain, only 19 items from the knowledge domain and 24 items from the stigma domain were retained after the CVI analysis.

**Table 1:** Content validation of knowledge domain

ITEM	EXPERTS				NUMBER IN AGREEMENT	I-CVI	UA	MEAN I-CVI	S-CVI/UA	S-CVI/AVE
	1	2	3	4						
1	X	X	X	X	4	1.00	1	1.00	1.00	1.00
2	X	X	X	X	4	1.00	1			
3	X	X	X	X	4	1.00	1			
4	X	X	X	X	4	1.00	1			
5	X	X	X	X	4	1.00	1			
6	X	X	X	X	4	1.00	1			
7	X	X	X	X	4	1.00	1			
8	X	X	X	X	4	1.00	1			
9	X	X	X	X	4	1.00	1			
10	X	X	X	X	4	1.00	1			
11	X	X	X	X	4	1.00	1			
12	X	X	X	X	4	1.00	1			
13	X	X	X	X	4	1.00	1			
14	X	X	X	X	4	1.00	1			
15	X	X	X	X	4	1.00	1			
16	X	X	X	X	4	1.00	1			
17	X	X	X	X	4	1.00	1			
18	X	X	X	X	4	1.00	1			
19	X	X	X	X	4	1.00	1			
Proportion relevance	1.00	1.00	1.00	1.00

**Table 2:** Content validation of stigma domain

ITEM	EXPERTS				NUMBER IN AGREEMENT	I-CVI	UA	MEAN I-CVI	S-CVI/UA	S-CVI/AVE
	1	2	3	4						
1	X	X	X	X	4	1.00	1	1.00	1.00	1.00
2	X	X	X	X	4	1.00	1			
3	X	X	X	X	4	1.00	1			
4	X	X	X	X	4	1.00	1			
5	X	X	X	X	4	1.00	1			
6	X	X	X	X	4	1.00	1			
7	X	X	X	X	4	1.00	1			
8	X	X	X	X	4	1.00	1			
9	X	X	X	X	4	1.00	1			
10	X	X	X	X	4	1.00	1			
11	X	X	X	X	4	1.00	1			
12	X	X	X	X	4	1.00	1			
13	X	X	X	X	4	1.00	1			
14	X	X	X	X	4	1.00	1			
15	X	X	X	X	4	1.00	1			
16	X	X	X	X	4	1.00	1			
17	X	X	X	X	4	1.00	1			
18	X	X	X	X	4	1.00	1			
19	X	X	X	X	4	1.00	1			
20	X	X	X	X	4	1.00	1			
21	X	X	X	X	4	1.00	1			
22	X	X	X	X	4	1.00	1			
23	X	X	X	X	4	1.00	1			
24	X	X	X	X	4	1.00	1			
Proportion relevance	1.00	1.00	1.00	1.00						

As shown in both Tables 1 and 2, all retained items in the knowledge domain and stigma domain of the WoCKSS received a CVI score of 1.00 from all four experts, indicating that they achieved perfect agreement among the panel of experts. The CVI score represents the proportion of experts who rated an item as relevant and is an indicator of the content validity of the scale. The maximum possible CVI score is 1.00, indicating that all experts rated an item as highly relevant.

The individual CVI (I-CVI) score for each item was calculated by dividing the number of experts who rated an item as relevant by the total number of experts. As shown in Table 1, all 19 items received an I-CVI score of 1.00, indicating that all experts rated all items as highly relevant. The mean I-CVI score, which represents the average of the I-CVI scores across all items, was also calculated and found to be 1.00, indicating excellent agreement among the experts regarding the relevance of the items in the knowledge domain.

The UA score represents the proportion of items that achieved a perfect CVI score of 1.00, while the S-CVI/AVE score represents the average CVI scores across all items. As shown in Table 1, all 19 items achieved a perfect CVI score of 1.00, resulting in a UA score of 1.00, indicating perfect agreement among the panel of experts regarding the relevance of the items in the knowledge domain. The S-CVI/AVE score was also found to be 1.00, indicating excellent content validity for the knowledge domain of the workplace COVID-19 knowledge scale in the manufacturing industry.

### Face validation

Table 3 presents the characteristics of the 10 respondents involved in the face validity assessment. The mean age of the respondents was 28 years old. Job positions of the respondents were diverse with 30% of them being operators or others, 20% were supervisors, and only 10% were executives. The majority of respondents had a monthly income of less than RM 2500, and 90% had a history of COVID-19 infection. All respondents reported having no chronic diseases and not smoking. All respondents had received at least two doses of COVID-19 vaccine, and 70% of them had a history of COVID-19 infection among family members.

**Table 3:** Characteristics of respondents involved in face validity (*n* = 10)

Variables	Mean (SD)	n (%)
Age	28.44 (8.11)	
Gender		
Female		8 (80.0)
Male		2 (20.0)
Education		
Lower secondary		1 (10.0)
Upper secondary		5 (50.0)
Pre-University		2 (20.0)
Tertiary		2 (20.0)
Job Position		
Executive		1 (10.0)
Supervisor		2 (20.0)
Operator		3 (30.0)
Others		3 (30.0)
Monthly income		
< RM 2500		7 (70.0)
RM 2501—RM 4850		3 (30.0)
COVID-19 infection history		
No		1 (10.0)
Yes		9 (90.0)
Chronic disease		
No		10 (100.0)
Yes		0
COVID-19 vaccination		
Not vaccinated		0
1 dose		0
At least 2 dose		10 (100.0)
History of COVID-19 infection among family members		
No		3 (30.0)
Yes		7 (70.0)
History of family members died due to COVID-19		
No		9 (90.0)
Yes		1 (10.0)
Smoking status		
Not smoking		10 (100.0)
Smoking		0

From an initial 19 items and 24 items in the knowledge and stigma domains, respectively, only 17 items from the knowledge domain and 22 items from the stigma domain were retained after FVI.

Tables 4 and 5 present the results of the Face Validity Index (FVI) for the knowledge and stigma domains. The FVI measures the level of agreement among respondents on the clarity and comprehensibility of each item in the domains, rated on a scale from 1 (not clear/comprehensible) to 4 (very clear/comprehensible). The tables include the number of respondents who agreed on the clarity and comprehension of each item (Number in Agreement), the Universal Agreement (UA) index, the Item-Level Content Validity Index (I-FVI), the mean I-FVI, the Scale-Level Content Validity Index/Average (S-FVI/AVE), and the Scale-Level Content Validity Index/Universal Agreement (S-FVI/UA).

**Table 4:** Face validation of knowledge domain

ITEM	RESPONDENTS										NUMBER IN AGREEMENT	UA	I-FVI	MEAN I-FVI	S-FVI/UA	S-FVI/AVE
	1	2	3	4	5	6	7	8	9	10						
1	X	X	X	X	X	X	X	X	X	X	10	1	1.00	0.99	0.88	0.88
2	X	X	X	X	X	X	X	X	X	X	10	1	1.00			
3	X	X	X	X	X	X	X	X	X	X	10	1	1.00			
4	X	X	X	X	X	X	X	X	X	X	10	1	1.00			
5	X	X	X	X	X	X	X	X	X	X	10	1	1.00			
6	X	X	X	X	X	X	X	X	X	X	10	1	1.00			
7	X	X	X	X	X	X	X	X	X	X	10	1	1.00			
8	X	X	X	X	X	X	X	X	X	X	10	1	1.00			
9	X	X	X	X	X	X	X	X	X	X	10	1	1.00			
10	X	X	X	X	X	X	X	X	X	X	10	0	1.00			
11	X	X	X	X	X	X	X	X	X	X	10	1	1.00			
12	X	X	X	X	X	X	X	X	X	X	10	1	1.00			
13	X	X	X	X	X	X	X	X	X	X	10	1	1.00			
14	X	X		X	X	X	X	X	X	X	9	0	0.90			
15	X	X	X	X	X	X	X	X	X	X	10	0	1.00			
16	X	X	X	X	X		X	X	X	X	9	0	0.90			
17	X	X	X	X	X	X	X	X	X	X	10	1	1.00			
Proportion relevance	0.89	0.89	0.84	0.89	0.89	0.84	0.89	0.89	0.89	0.89

**Table 5:** Face validation of stigma domain

ITEM	RESPONDENTS										NUMBER IN AGREEMENT	UA	I-FVI	MEAN I-FVI	S-FVI/UA	S-FVI/AVE
	1	2	3	4	5	6	7	8	9	10						
1	X	X	X	X	X	X	X	X	X	X	10	1	1.00	0.99	0.86	0.90
2	X	X	X	X	X	X	X	X	X	X	10	1	1.00			
3	X		X	X	X	X	X	X	X	X	9	0	0.90			
4	X	X	X	X	X	X	X	X	X	X	10	1	1.00			
5	X	X	X	X	X	X	X	X	X	X	10	1	1.00			
6	X		X	X	X	X	X	X	X	X	9	0	0.90			
7	X	X	X	X	X	X	X	X	X	X	10	1	1.00			
8	X	X	X	X	X	X	X	X	X	X	10	1	1.00			
9	X	X	X	X	X	X	X	X	X	X	10	1	1.00			
10	X	X	X	X	X	X	X	X	X	X	10	1	1.00			
11	X	X	X	X	X	X	X	X	X	X	10	1	1.00			
12	X	X	X	X	X	X	X	X	X	X	10	1	1.00			
13	X	X	X	X	X	X	X	X	X	X	10	1	1.00			
14	X	X	X	X	X	X	X	X	X	X	10	1	1.00			
15	X		X	X	X	X	X	X	X	X	9	0	0.90			
16	X	X	X	X	X	X	X	X	X	X	10	1	1.00			
17	X	X	X	X	X	X	X	X	X	X	10	1	1.00			
18	X	X	X	X	X	X	X	X	X	X	10	1	1.00			
19	X	X	X	X	X	X	X	X	X	X	10	1	1.00			
20	X	X	X	X	X	X	X	X	X	X	10	1	1.00			
21	X	X	X	X	X	X	X	X	X	X	10	1	1.00			
22	X	X	X	X	X	X	X	X	X	X	10	1	1.00			
Proportion relevance	0.92	0.79	0.92	0.92	0.92	0.92	0.92	0.92	0.92	0.92						

For the knowledge domain, the results indicate that among the retained 17 items, most items received high levels of agreement, with thirteen out of seventeen items achieving a UA index of 1.00. The mean I-FVI was also high, indicating that most items were considered highly relevant. The S-FVI/UA was 0.89, indicating a high level of agreement among respondents on the overall clarity and comprehensibility of the scale.

For the stigma domain, the results of the retained 22 items show that most of the items received a high level of agreement on their relevance, with 19 items having a unanimous agreement among the participants. The overall mean I-FVI score for the STIGMA domain is 0.99, and the S-FVI/UA and S-FVI/AVE values are 0.86 and 0.90, respectively. These results suggest that the stigma domain is well-defined and that the items included in the FVI are clear and comprehensible to the respondents.

## Discussion

WoCKSS represents a significant step forward in addressing the critical need for comprehensive tools to assess COVID-19 knowledge and stigma within workplace settings, particularly in the manufacturing industry. While numerous scales have been developed for assessing COVID-19 stigma [20, 21, 28, 29], WoCKSS stands out due to its tailored approach designed specifically for the nuances of the Malaysian manufacturing sector. The development of WoCKSS meticulously considered the distinctive cultural, socioeconomic, and educational diversity prevalent within the Malaysian manufacturing workforce. This localization effort is underscored by the decision to construct WoCKSS in the Malay language, the national language of Malaysia, ensuring linguistic appropriateness and reinforcing cultural relevance. The localization process, validated through face validity, introduces a unique dimension to WoCKSS, rendering it finely attuned to the intricacies of the Malaysian manufacturing setting. By aligning with the linguistic and cultural characteristics of the target population, WoCKSS emerges as a more fitting and contextually relevant instrument for assessing COVID-19 knowledge and stigma in the Malaysian manufacturing sector.

This instrument was developed by taking into considerations the three constructs that make up stigma: stereotype, fear and prejudice [20]. The integration of these constructs into the WoCKSS ensures a more comprehensive assessment of COVID-19-related stigma in the Malaysian workplace. In the workplace setting, 'stereotyping' can give rise to discrimination based on perceived COVID-19 status, while 'prejudice' may foster biased attitudes and unequal treatment of affected individuals. The presence of 'fear' can induce anxiety and avoidance behaviors, impacting both workplace safety and efficiency [30]. These manifestations of stigma in the context of COVID-19 not only affect individual well-being but also have wider implications for workplace efficiency and safety. Addressing these specific manifestations is pivotal for the development of effective interventions aimed at reducing stigma and improving the overall workplace environment. WoCKSS holds practical applications in employee training programs to assess and enhance COVID-19 knowledge for a safer workforce. Moreover, its stigma domain can guide interventions to reduce discriminatory behaviors and foster inclusivity. The scale's potential impact on policy and workplace practices is noteworthy, offering insights for the development of targeted policies and enabling employers to tailor practices, including educational campaigns and support systems addressing stigma-related issues.

Involvement of experts supported by the results of the content validity assessment demonstrate that the items in both knowledge and stigma domain of WoCKSS are relevant for measuring knowledge and stigma related to COVID-19 in the manufacturing industry. The high level of agreement among experts on the relevance of the items in the knowledge and stigma domains suggests that the domains are well-defined and include highly relevant items that accurately capture the knowledge and stigma-related issues associated with COVID-19 in the workplace. These findings are consistent with previous research, which highlights the importance of assessing content validity to ensure that an instrument accurately measures the construct of interest. For instance, studies on the assessment of the reliability of the Arabic version of the Corona Virus Anxiety Scale (CAS) and the impact of home confinement during the COVID-19 pandemic on sleep in subjects with Parkinson's disease which also reported high CVI [31, 32].

For face validity, the selected respondents represented various job positions within the manufacturing setting. This distribution captured perspectives from different hierarchical levels within the workplace, recognizing that knowledge and stigma experiences may vary across roles. Moreover, the respondents exhibited diverse educational levels, reflecting the educational spectrum within the manufacturing workforce. The variation in educational backgrounds among respondents ensures a nuanced understanding of COVID-19 knowledge and stigma across different levels of educational attainment. Therefore, these factors reinforcing the applicability and generalizability of the Workplace COVID-19 Knowledge and Stigma Scale (WoCKSS) to the wider manufacturing workforce in Malaysia.

The findings from the face validity assessment indicate that the items remained in the WoCKSS are acceptable and can be understand by the target population in the manufacturing industry. The high level of agreement among respondents on the clarity and comprehension of most items in both the knowledge and stigma domains indicates that the WoCKSS is easy to be used for assessing COVID-19 knowledge and stigma in the workplace. These findings align with previous research demonstrating high FVI, underscoring the importance of assessing face validity as an essential step in instrument development. This is exemplified in a study on COVID-19-related stigma among the general population in Iran [33].

The robustness of any psychometric instrument lies in its ability to accurately measure the intended constructs. In this study, the CVI and FVI were pivotal in establishing the credibility and appropriateness of the Workplace COVID-19 Knowledge and Stigma Scale (WoCKSS) within the context of the manufacturing workforce in Malaysia.

The CVI, calculated based on expert ratings, represents the proportion of experts who rated each item as relevant, with a maximum possible score of 1.00 indicating unanimous agreement among experts [22]. In our study, all retained items achieved a CVI score of 1.00, demonstrating perfect agreement among the panel of experts. This unanimous consensus among experts with diverse backgrounds in public health, microbiology, and primary care medicine signifies a high level of agreement on the relevance, clarity, and appropriateness of the WoCKSS items for measuring workplace COVID-19 knowledge and stigma.

Furthermore, the FVI, derived from respondent ratings on clarity and comprehension, adds an additional layer of validation. It is important because it ensure that the target population interpreted the items in the same way as intended by the scale developers [25, 34].This aspect is crucial in ensuring that the scale is not only for expert endorsement but also for ensuring the scale’s understandability and relevance to the target population [26]. Our study achieved high FVI scores, indicating strong agreement among respondents on the clarity and comprehensibility of the WoCKSS items. This outcome is particularly noteworthy given the diverse job positions and educational backgrounds of the respondents.

While the statistical significance of CVI and FVI is not conventionally discussed in the same way as inferential statistics, such as p-values, the perfect agreement scores (CVI and FVI of 1.00) should not be understated. These scores not only attest to the relevance and clarity of the items but also underscore the methodological rigor employed in the scale development and validation process. The unanimity among both experts and respondents adds a layer of confidence in the reliability and validity of the WoCKSS for assessing COVID-19 knowledge and stigma in the manufacturing industry.

It is important to explicitly acknowledge the limitations of the study. Firstly, the sample size for face validation was relatively small, involving 10 respondents from a single manufacturing company. While this limited sample was chosen due to logistical constraints, it aligns with the minimum acceptable number of raters according to reference standards [26]. Nevertheless, it is recognized that many studies opt for a larger sample size, often reaching 30 raters, which could provide a more comprehensive validation of the face validity [26]. Therefore, the findings should be interpreted with caution, considering the potential limitations associated with the sample size. Secondly, the potential for biases in expert selection should be recognized. The panel of experts encompassed individuals with expertise in public health, microbiology, and primary care medicine. While these experts brought valuable insights from their respective fields, the absence of direct experience in COVID-19-related scales might be considered a limitation. However, it is crucial to note that these experts work closely with COVID-19-related issues within their broader expertise, and the development of WoCKSS emerged from the necessity of having a Malay-language questionnaire specific to COVID-19 stigma, which was not previously available.

Although CVI and FVI are important validity tests, they alone are not sufficient to establish the construct validity of WoCKSS. Construct validity refers to the degree to which a scale measures the intended construct or concept. To establish this construct validity, WoCKSS needs to undergo additional analyses such as exploratory factor analysis (EFA) and confirmatory factor analysis (CFA) on bigger sample populations. Exploratory factor analysis is used to identify the underlying structure or factors of a scale, while confirmatory factor analysis is used to confirm the factor structure identified through EFA. These analyses ensure that the items included in the scale are measuring the intended construct [25]. Therefore, the findings from this study provide an important first step in the development and validation of WoCKSS. It is important to note that construct validity of WoCKSS is still ongoing and will be made available in the future as more data becomes available.

## Conclusion

In conclusion, the Workplace COVID-19 Knowledge and Stigma Scale (WoCKSS) has demonstrated promising content and face validity in assessing COVID-19 knowledge and stigma within the manufacturing industry. The robust agreement among experts and respondents indicates that the scale has the potential to be a valuable tool for measuring COVID-19-related knowledge and stigma in the workplace. The development of the WoCKSS serves as a culturally relevant and context-specific instrument tailored to the unique challenges faced by the manufacturing workforce in Malaysia. By focusing on the manufacturing sector, this study recognizes the necessity of industry-specific tools to comprehensively assess knowledge and stigma related to COVID-19.

## Data Availability

The data used in this study are available and will be provided by the corresponding author on a reasonable request.
